# Rhino-orbito-cerebral Mucormycosis: Pictorial Review

**DOI:** 10.1186/s13244-021-01109-z

**Published:** 2021-11-12

**Authors:** Vivek Pai, Rima Sansi, Ritesh Kharche, Sridevi Chaitanya Bandili, Bhujang Pai

**Affiliations:** 1 Department of Radiology, SevenHills Hospital, Mumbai, India; 2 Department of Histopathology, SevenHills Hospital, Mumbai, India; 3 Department of Microbiology, SevenHills Hospital, Mumbai, India

**Keywords:** Mucormycosis, Rhino-orbito-cerebral Mucormycosis, Invasive fungal sinusitis

## Abstract

Mucormycosis (MCR) is a fulminant, potentially lethal, opportunistic fungal infection. Diabetes, immunocompromised states and elevated serum iron levels are the most important risk factors for contracting MCR infection. Recently, MCR co-infections have been observed in patients with COVID-19 disease owing to a complex interplay of metabolic factors and corticosteroid therapy. Rhino-orbito-cerebral mucormycosis (ROCM) is the most common clinical form of MCR infection and refers to infection of the nasal cavities, paranasal sinuses, neck spaces, orbits and intracranial structures. Sinonasal inoculation is typically the primary site of infection; the necrotising and angioinvasive properties of the fungus facilitate its spread into adjacent structures. In this review, we discuss the pertinent mycology and risk factors of MCR infection. The review also aims to acquaint the reader with the cross-sectional imaging appearances of ROCM and its complications. All the cases discussed in this pictorial essay are microbiologically and/or histopathologically proven cases of ROCM with concomitant COVID-19 infection.

## Key points

- Rhino-orbito-cerebral Mucormycosis is potentially fatal; mortality depends on the extent of spread.
- Cross-sectional imaging provides quick, presumptive diagnosis and guides management.
- The “black turbinate” sign is a common imaging finding, but is not specific.
- Angioinvasion facilitates rapid extra-sinus spread without overt bone destruction.
- Perineural spread constitutes an important pathway for intracranial spread.

## Background

**Fig. 1:**
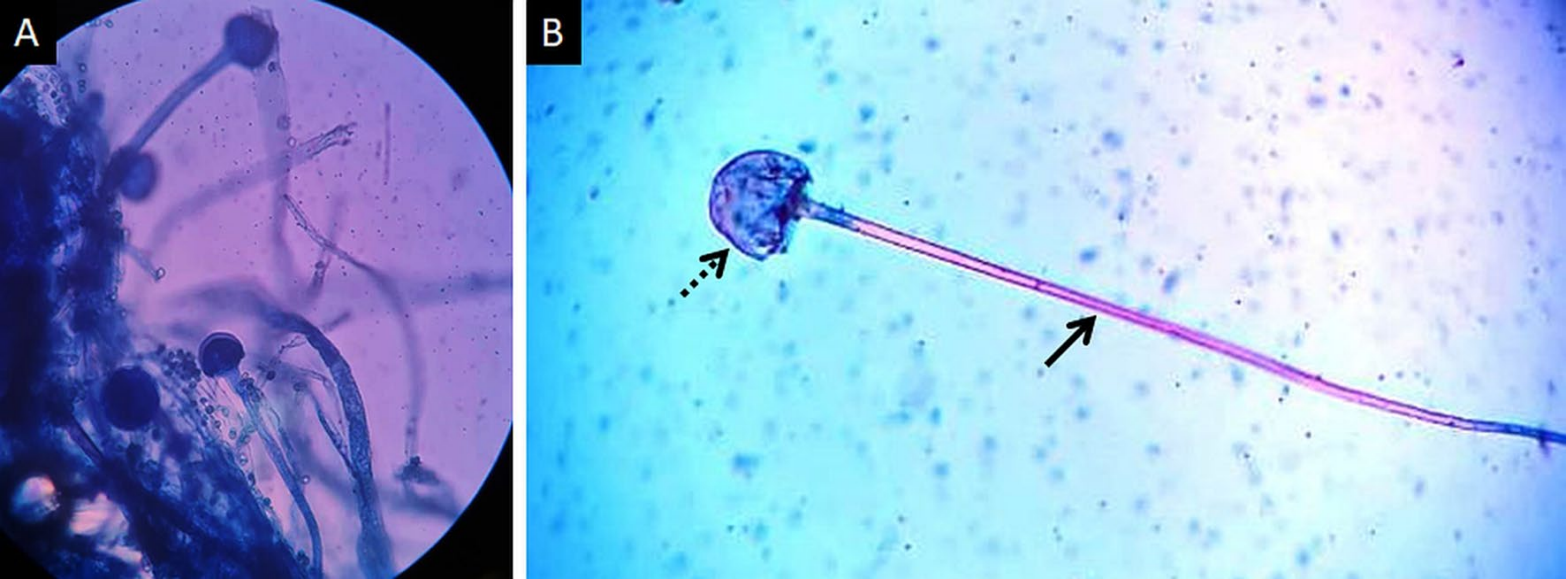
Lactophenol cotton blue (LCB) stained tissue mount (**a**) performed for the demonstration of sporangiophores, the early stage in the life cycle of MCR. High-power photomicrograph (**b**) of a single sporangiophore (thick arrow) with a bulbous, sac-like sporangium (dotted arrow) containing new fungal spores

Mucormycosis (MCR) is an opportunistic infection caused by fungi belonging to order Mucorales [1]. Frequently reported species belong to genera Rhizopus, Lichtemia and Mucor [2]. These fungi are saprophytic and commonly found in soil, decaying food and excrements [3] (Fig. 1). Human infection mainly occurs due to inhalation of spores; ingestion of contaminated food and traumatic inoculation are uncommon modes of transmission [4]. The hallmark of MCR infection is angioinvasion leading to vascular thrombosis, widespread tissue necrosis and systemic dissemination [5].

### Risk factors

Diabetes mellitus (DM) is a major predisposing factor in MCR infection. DM reduces immunity making patients susceptible to infection [6]. The incidence of MCR in patients with DM ranges between 17% and 88% globally [4]. In fact, 70% patients with ROCM are found to have underlying DM [7]. Elevated free serum iron and deferoxamine chelation therapy are also known to increase the risk of MCR [5, 7].

MCR is also common among patients with neutropenia. 2%–15% cases of MCR are seen in patients with solid organ malignancies and organ transplants [4]. Immunosuppressive therapies, especially high-dose steroid (≥ 600 mg prednisone), used in the management of malignancies, transplantation and autoimmune conditions, also increase the risk of MCR [4, 8]. Patients who receive prophylactic antifungal therapy have been identified as an important risk group for MCR [4, 8]. Breakthrough MCR was first reported with voriconazole prophylaxis and subsequently with other triazoles [4, 8]. Voriconazole is a broad spectrum antifungal that does not inhibit Mucorales [7]. The inhibition of other fungi and overall improved life expectancy of immunosuppressed patients have been hypothesised to increase risk of MCR infection [7].

Other risk factors include HIV infection, intravenous drug abuse, malnutrition, chronic alcoholism, liver disease and treatment with calcineurin inhibitors [4]. MCR may occur in immunocompetent patients suffering from burns, following major trauma or through contaminated surgical material [4].

### COVID-19 and the risk of Mucormycosis

The coronavirus disease 2019 (COVID-19) is caused by the novel severe acute respiratory syndrome coronavirus 2 (SARS-CoV-2). While most of the clinical manifestations and complications of COVID-19 have been documented with standardised treatment protocols, multiple fungal opportunistic infections have now emerged as the new threat to a favourable prognosis. Of these a steady surge of MCR co-infections has been observed leading to the coinage of the term COVID-19-associated Mucormycosis (CAM) [9, 10].

Corticosteroids used in the management of COVID-19 have been identified as a potential risk factor for MCR infection, due to the induced reduction of immune function and altered glucose homeostasis. In addition severe COVID-19 disease has been associated with a hyperferritinemic syndrome increasing the risk of cellular damage and increased free iron ultimately increasing the risk of MCR [11, 12]. The rampant use of supplements containing zinc and iron, both nutrients for Mucorales, has also been speculated in the rise of CAM. Sen et al. suggested that ROCM occurred in patients with severe COVID-19 disease, those admitted to the intensive care unit or requiring mechanical ventilation [13]. Banerjee et al. proposed that the inadvertent use of industrial oxygen, reusable oxygen humidifiers and poor sanitation may also contribute to ROCM in COVID-19 patients.

### Rhino-orbito-cerebral mucormycosis (ROCM)

ROCM describes a fulminant infection of the nasal cavities, paranasal sinuses, neck spaces, orbits and intracranial structures caused by Mucorales species. ROCM presents in an acute setting, similar to sinusitis [14]. Fever, facial pain, facial swelling, headache, nasal discharge, nasal ulceration and palatal ulceration are common presenting features [15]. Foul smelling, black, necrotic eschar of the nasal mucosa or palate is seen in about 50% cases, but is a sentinel sign of ROCM [14, 16]. This necrotic black tissue occurs due to local vascular thrombosis and tissue infarction [14]. Orbital involvement may vary between 66% and 100% [17]. Manifestations of orbital spread include periorbital swelling, palpebral abscess, orbital cellulitis, proptosis, chemosis, reduced vision, vitritis, endophthalmitis, orbital apex syndrome and ophthalmoplegia [15]. Blindness, a major complication of ROCM, may result from optic nerve infarction due to central retinal artery/ophthalmic artery occlusion or involvement of the orbital apex [17]. Intracranial progression may occur by direct extension or angioinvasion, usually within the span of days [18]. Nearly all patients have orbital involvement by the time intracranial disease is diagnosed [15]. Intracranial spread is heralded by signs of cavernous sinus thrombosis, hemiparesis, altered mentation and focal seizures [16].

### Diagnosis

**Fig. 2:**
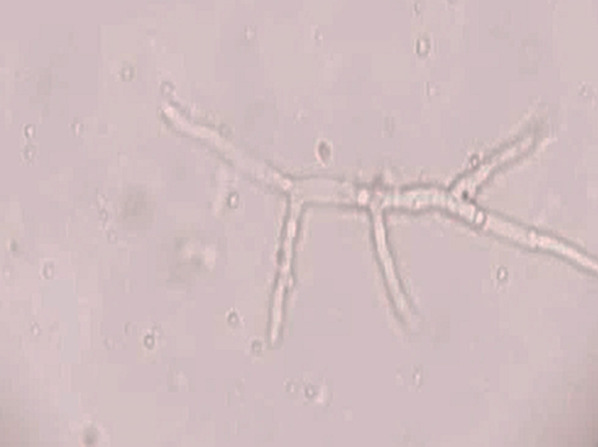
High-power microscopy of a potassium hydroxide (KOH) mount demonstrates a broad, aseptate, branching (at 90◦) hypha of Mucorales

**Fig. 3:**
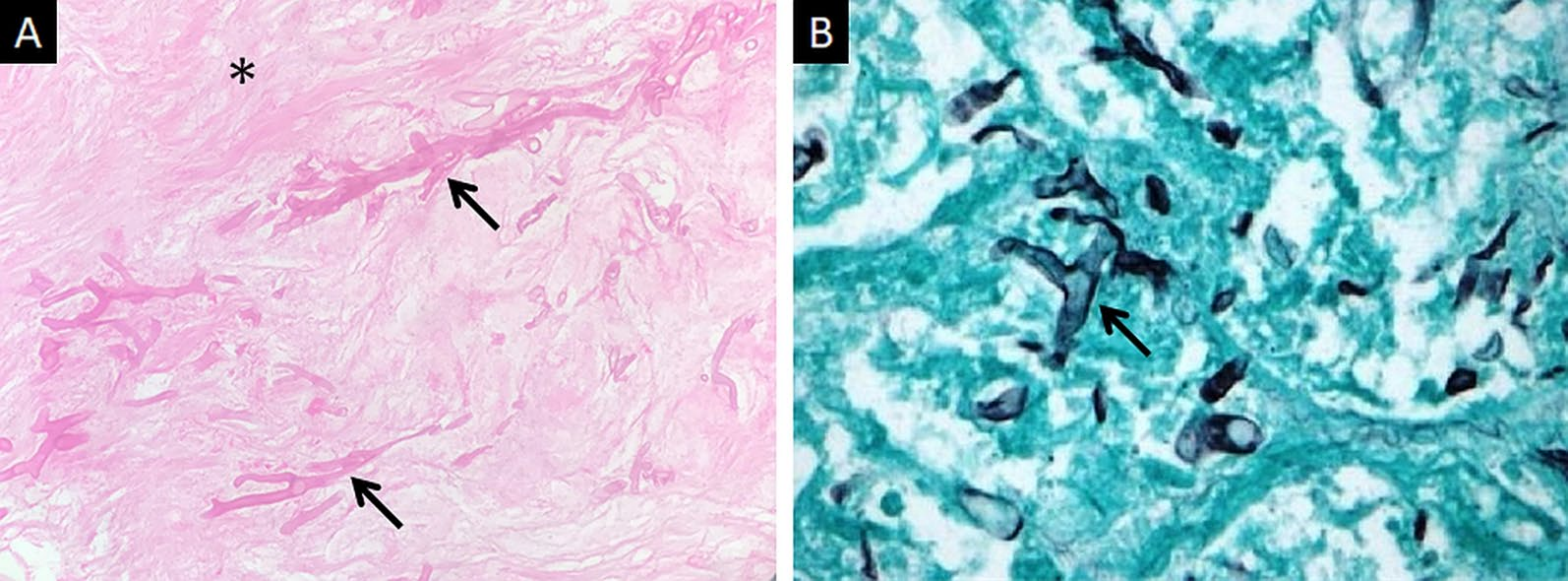
Histopathological examination of neck tissues following surgical debridement of 2 different patients with ROCM. Haematoxylin and Eosin (HE) staining (**a**) demonstrates broad, branching fungal hyphae (thick arrows) on the background of extensive tissue necrosis (*). Grocott-Gomori methamine silver staining (**b**) relies on oxidation of the polysaccharides within fungal cell walls. The result is black staining of hyphae (thick arrow in **b**) against a green counterstain

**Fig. 4:**
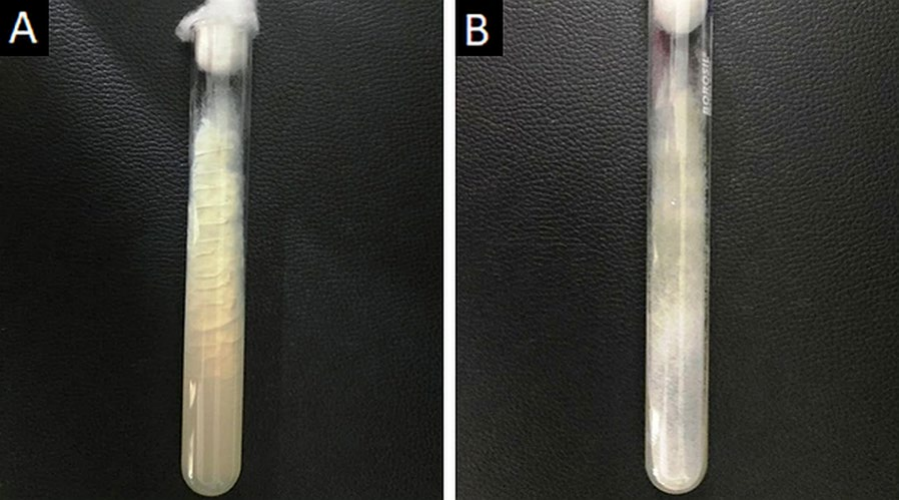
Obverse (**a**) and reverse (**b**) views of Sabouraud Dextrose Agar used for the selective cultivation of Mucorales species at 30◦ C. Note the greyish white, cotton-like appearance of the fungal colonies

Direct microscopy, histopathology and fungal culture of surgical specimens form the cornerstones in the diagnosis of MCR. Hyphae of Mucorales demonstrate a broad (6–25 μm), non-septate, branching (90◦ bifurcations) morphology and can be detected using direct microscopy or on a potassium hydroxide (KOH) wet mount preparation (Fig. 2) [19]. Neutrophilic or granulomatous inflammation is the predominant finding on histopathology. Prominent necrosis and angioinvasion are common [19] (Fig. 3). Histopathological tissue diagnosis is still one of the most accurate and fast diagnostic modalities for MCR. All Mucorales species grow rapidly [3–7 days] on Sabouraud dextrose agar at a temperature of 25–30 °C [19] (Fig. 4). Recently introduced molecular assays, using polymerase chain reaction (PCR)-based techniques, aid in rapid diagnosis and speciation of Mucorales from a variety of specimen [8].

### Treatment

Effective treatment of ROCM requires high index of clinical suspicion, expeditious laboratory and radiological diagnosis as well as a multidisciplinary management. Antifungal therapy using 5–10 mg/kg of liposomal amphotericin B, aggressive surgical debridement and elimination of the underlying risk factors are key in the effective management of ROCM [20]. Surgical management includes extensive debridement along with turbinectomy, palatal resection, lamina papyracea resection and occasionally orbital exenteration. Retro-bulbar injection and/or sinus irrigation with 3.5 mg/ml and 1 mg/ml, respectively, using amphotericin B may be performed. Following clinical and radiological stabilisation or regression of the disease, medical management may be subsequently stepped down to oral isavuconazole or oral posaconazole [20].

### Prognosis

Prognosis of ROCM is dismal, even with aggressive surgical and medical treatment. Mortality of ROCM varies between 25% and 80% and depends of on the extent of the disease, promptness of treatment and underlying risk factors. In a prospective study by Chamilos et al., the survival rate was found to be 85% when treatment was initiated within 5 days of diagnosis as compared to 49% when treatment was started following the 6th day [21]. Combination of surgical and medical therapy has been associated with better survival (70%) as compared to only surgical management (57%) or only antifungal chemotherapy (61%) [21].

As the situation of concomitant ROCM and COVID-19 still evolves, it is difficult to ascertain the exact mortality rate. The mortality rate of CAM in general is estimated to range between 28 and 52% in different studies [10]. A more extensive case series would be necessary to estimate the mortality of ROCM–COVID-19 co-infection.

## Imaging of Rhino-orbito-cerebral Mucormycosis

CT and MRI help in making an early pre-operative diagnosis of ROCM. Imaging plays a vital role in evaluation of disease spread which in turn guides medical and surgical management. CT is quick and readily available; common findings include opacification of the sinonasal air spaces and obliteration of the deep neck fat pads. Bone destruction, if any, is best identified on CT bone algorithms. MRI, with its excellent inherent soft tissue resolution, helps in characterising sinonasal contents, involvement of the deep neck soft tissues and spaces as well as identify perineural and intracranial spread. In an appropriate clinical setting, the imaging features of ROCM are diagnostic [22].

For the ease of reporting, we classify ROCM into 2 broad categories, based on anatomic involvement: sinonasal (nasal cavity and paranasal sinuses) and extra-sinus (deep neck, orbital and intracranial) involvement. A detailed account of the previously reported literature and pertinent imaging features of ROCM is presented.

### Sinonasal involvement

ROCM typically begins in the nasal mucosa and spreads into the paranasal sinuses [23]. CT findings of nasal MCR are non-specific and sparsely reported. In a study to characterise CT findings in invasive fungal sinusitis (IFS) (mucormycosis, aspergillosis), DelGaudio et al. found severe thickening of the nasal mucosa, turbinates, septum and nasal floor in 91% (21/23) patients [24]. CT may also reveal isolated, non-specific turbinate hypertrophy with/without inflammatory fluid [2]. Nasal septal perforation has been identified by Therakathu et al. in ROCM [2].

**Fig. 5:**
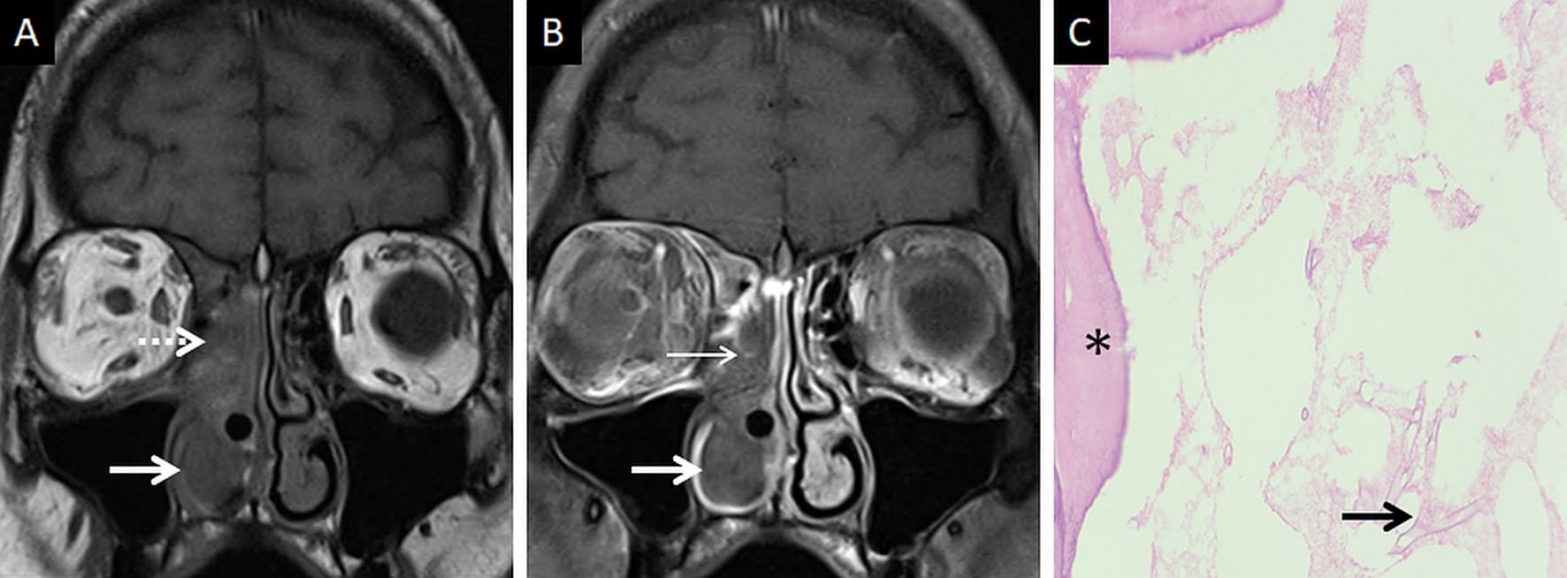
Classic appearance of the “black turbinate” sign. Unenhanced coronal T1W image (**a**) reveals intermediate signal contents within the right nasal cavity (dotted arrow) and hypertrophy of the right inferior turbinate (thick arrow). Corresponding CE fat-suppressed (FS) coronal T1W image (**b**) reveals poor enhancement of the right middle (thin arrow) and inferior turbinates (thick arrow) due to necrosis, resulting in the “black turbinate” sign. Post-operative histopathology analysis (**c**), using HE staining, demonstrates necrosis of the turbinate (*) and branching, aseptate fungal hyphae (thick arrow) consistent with the diagnosis of MCR

At MRI, the involved nasal mucosa may demonstrate varied signals, including hyperintensity (necrosis) or hypointensity (fungal paramagnetic material) on T2W images [2, 25]. Safder et al. reported 2 cases with restricted diffusion, in the turbinates and mucoperiosteal thickening within the maxillary sinus, representing infarcted tissue [25]. Necrosis and devitalisation of the turbinates may cause lack of contrast enhancement on MRI. This appearance is termed as the “black turbinate (BT) sign” [25] (Fig. 5). The BT sign was first described in nasal MCR, but subsequently reported in other cases of IFS [25, 26].

It is worthwhile to note that this imaging appearance has been identified in normal subjects as well [33]. The lack of enhancement in normal subjects is linked to the normal cavernous tissues within the turbinates [27]. Involvement of the posterior portions of the inferior and middle turbinates, well-defined margins, hyperintense T2 signals, thin peripheral enhancement, progressive enhancement on dynamic contrast-enhanced scans and T2 hyperintensity favour benign BT [27]. On the contrary, fungal BTs are confluent, ill defined, non-enhancing masses commonly involving the middle turbinate (because they filter majority of nasal airflow) [27, 28].

Involvement of the paranasal sinuses is common. In studies on ROCM, Bhansali et al., Ferry et al. and Yohai et al. reported PNS involvement in 100%, 69% and 79% patients, respectively [23, 29, 30]. Some authors identify the maxillary sinus to be most frequently involved; others suggest the ethmoid sinuses [2, 22]. Involvement of multiple sinuses is common; maxillary, ethmoid and sphenoid sinuses being a common combination [2]. In a study on 43 patients with proven ROCM by Therakathu et al., unilateral sinus involvement was found to be more common; however, in another study by Slonimsky et al. bilateral sinonasal involvement was found more in ROCM than aspergillosis [2, 31].

**Fig. 6:**
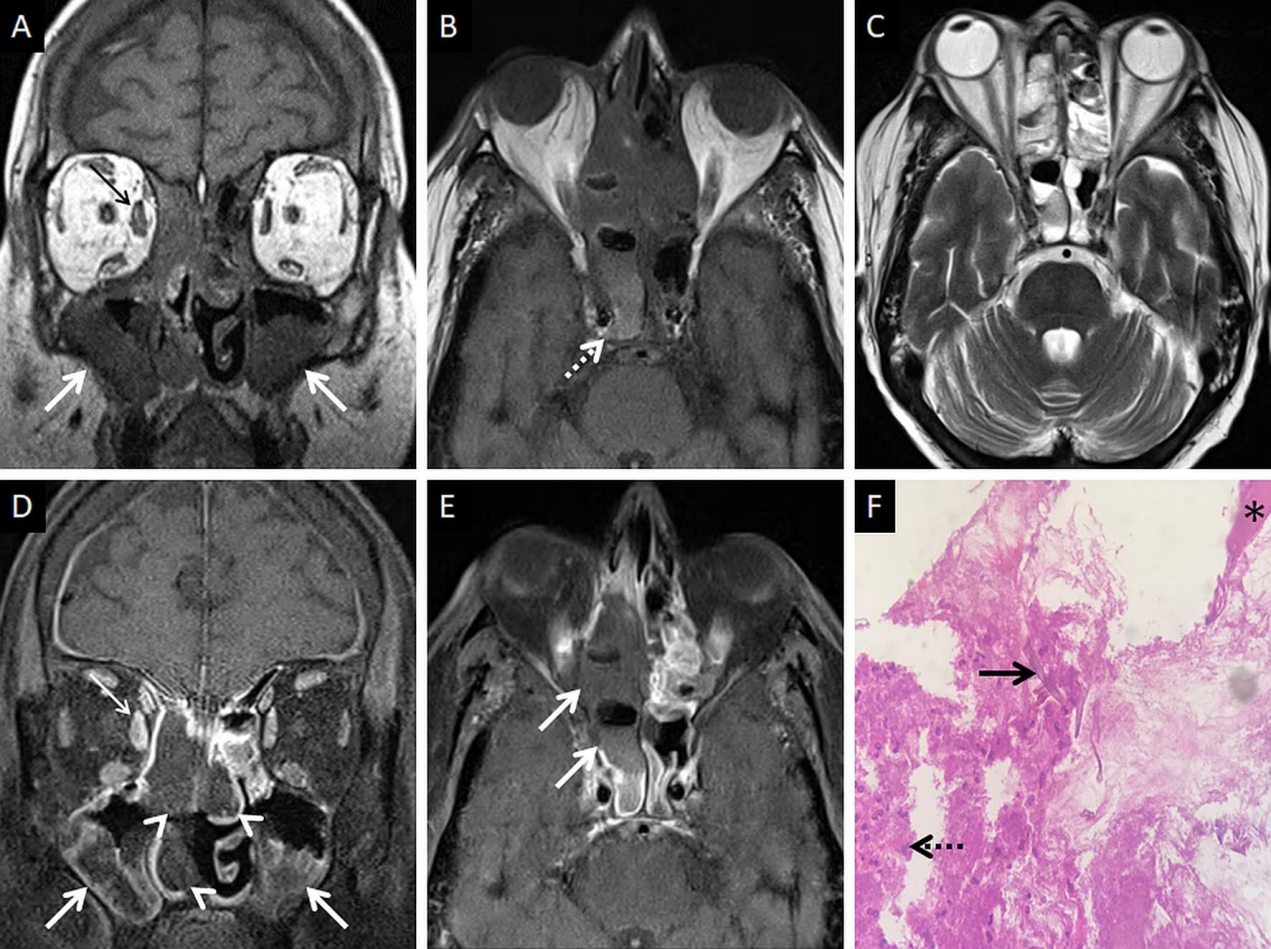
Sinonasal involvement of ROCM. Unenhanced coronal T1W image (**a**) reveals diffuse, intermediate signal intensity, mucosal and mucoperiosteal thickening (thick arrows) in the nasal cavities and the maxillary sinuses, respectively. Note the slight asymmetric thickening of the right medial rectus muscle (thin arrow). Unenhanced axial T1W image (**b**) reveals mucoperiosteal thickening in the ethmoid and sphenoid sinuses with mild T1 shortening in the right sphenoid sinus (dotted arrow), consistent with fungal paramagnetic content. Axial T2W image (**c**) reveals non-specific, mixed signal intensity signals within the ethmoid and sphenoid sinuses. CE FS coronal T1W image (**d**) shows lack of enhancement in both middle turbinates and the right inferior turbinate (black turbinate sign—arrow heads), along with non-enhancing mucoperiosteal thickening in the maxillary sinuses (thick arrow). Note the asymmetric enhancement of the right medial rectus muscle (thin arrow) suggesting myositis. CE FS axial T1W image (**e**) confirms poor enhancement of the right ethmoid and sphenoid sinus mucoperiosteal thickening (thick arrows). Post-operative histopathology analysis (**f**), using HE staining, demonstrates shows few branching, aseptate fungal hyphae (thick arrow) consistent with the diagnosis of MCR along with neutrophilic and histiocytic inflammatory infiltrates (dotted arrow) and necrotic tissues (*)

CT features of paranasal sinus disease in ROCM include a rind of soft tissue, dense opacification or variable, nodular, mucoperiosteal thickening [28, 31]. The presence of air fluid levels have been disputed in the literature [22, 25, 32]. Radiodense concretions, common in aspergillosis, are not seen in ROCM [33]. MRI signals of the sinus contents may vary, ranging from hyperintensity to profound hypointensity (due to fungal paramagnetic contents) on T2W images [22, 32] Contrast-enhanced scans also reveal range of enhancement patterns, including absent to heterogeneous intense enhancement [2] (Fig. 6). Bacterial sinusitis lacks local necrosis, hence, the mucosal T2 signals and enhancement are not affected [34].

### Extra-sinus spread

**Fig. 7:**
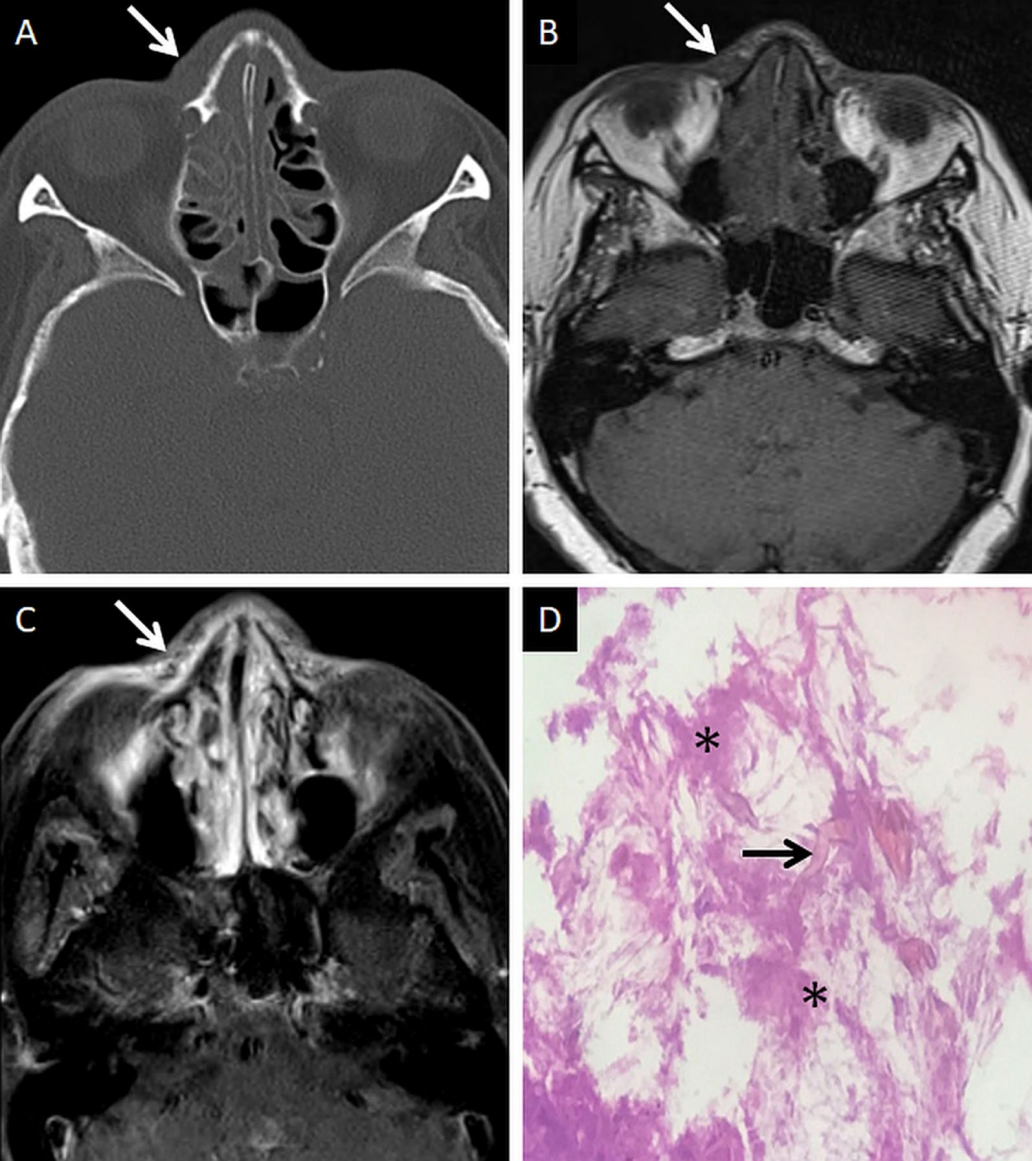
Extra-sinus involvement without bone destruction. Axial CT scan (**a**) of the paranasal sinuses, obtained using the bone algorithm, reveals opacification of the ethmoid air cells, rarefaction of its trabeculae in addition to subtle swelling along the nasal bridge (thick arrow). No overt erosion of the nasal bones, nasolacrimal duct or the lamina papyracea is seen. Unenhanced axial T1W image (**b**) confirms the subcutaneous soft tissue (thick arrow) detected on the CT. CE FS axial T1W image (**c**) demonstrates heterogeneous enhancement of the nasal soft tissue (thick arrow). Post-debridement histopathology analysis (**d**), using HE staining, confirms branching, aseptate fungal hyphae (thick arrow), consistent with MCR, on the background of necrotic tissue (*)

Extra-sinus extension of the MCR is common, often without radiological evidence of bony sinus wall destruction [32]. Bone destruction in the ROCM has been found in only 40% cases [16]. The angioinvasive propensity of the fungus, and its ability to disseminate along the perivascular channels, allows the invasion of adjacent structures through intact bony partitions [35] (Fig. 7). If involved, the bones may show rarefaction, erosions or permeative destruction on a CT scan [2].

### Deep neck involvement

**Fig. 8:**
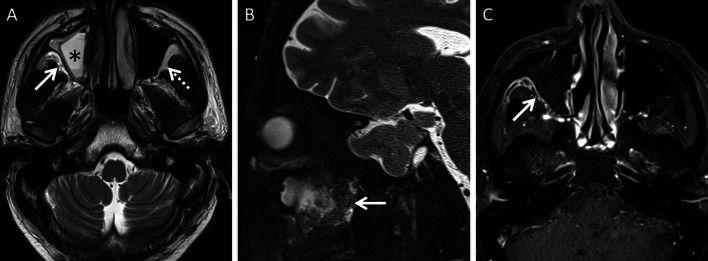
Retro-antral fat involvement in a patient with histopathologically confirmed MCR. Axial T2W image of the brain (**a**) reveals right maxillary sinus disease (*). Note the slightly heterogeneous appearance of the right retro-antral fat (thick arrow) as compared to the contralateral side (dotted arrow). Sagittal T2-FS image (**b**), obtained for the evaluation of the neck and orbits, confirms stranding of right retro-antral fat (thick arrow). CE FS axial T1W image (**c**) demonstrates intense enhancement of the right retro-antral soft tissue (thick arrow)

Stranding and/or soft tissue obliteration of the fat planes are important signs of fungal spread into the deep neck spaces [36]. Involvement of the distinct fat planes anterior and posterior to the maxillary sinus (anterior periantral fat and posterior periantral/retro-antral fat, respectively) is common. Retro-antral fat involvement is considered to be one of the initial imaging signs of deep neck invasion in ROCM [37, 38] (Fig. 8). Stranding or soft tissue in the periantral region is thought to be due to congestive oedema secondary to vascular thrombosis or the presence of fungal elements due to spread along blood vessels and perivascular spaces across the confines of the maxillary sinus [37]. Irrespective of the nature of the soft tissue, this region is an important review area, especially if dedicated CT/MRI of the neck is not performed.

The pterygopalatine fossa (PPF) may also be involved, due to spread from the nasal cavity via the sphenopalatine foramen [39]. Middlebrooks et al. suggested that involvement of the PPF correlated strongly with acute IFS (r = 0.64) and sphenopalatine foramen involvement was present in 72% of patients [40].

**Fig. 9:**
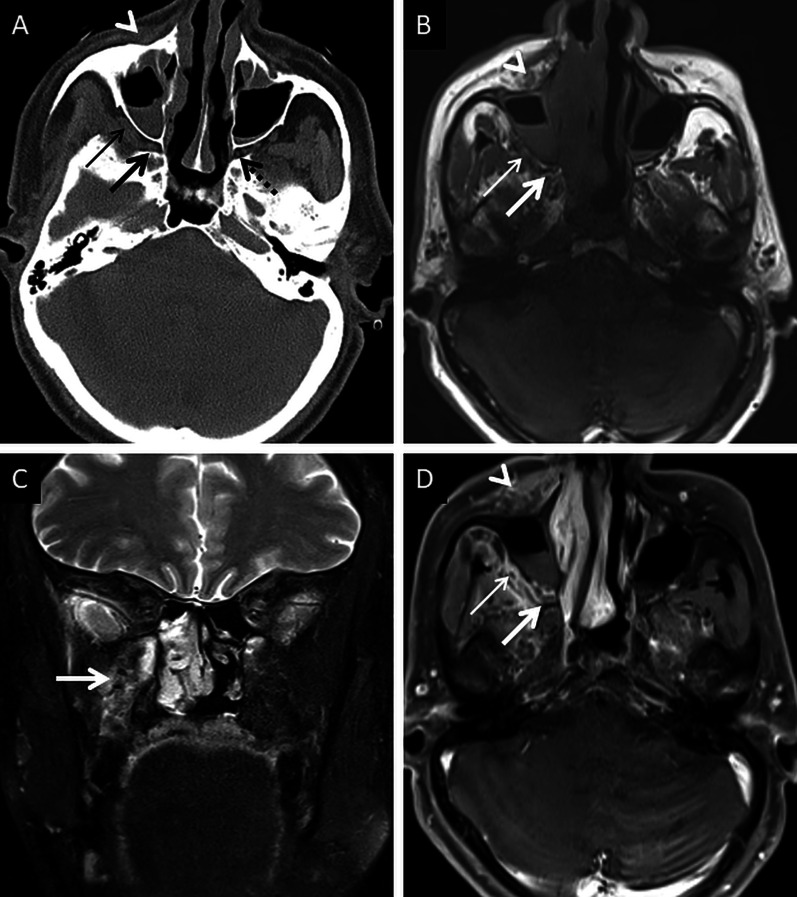
PPF involvement in ROCM. Unenhanced axial CT scan of the brain (**a**) reveals soft tissue within the right PPF (thick arrow). Note the normal fat density and branches of the internal maxillary artery seen in the contralateral, uninvolved PPF (dotted arrow). Soft tissue is also noted in the right retro-antral fat (thin arrow) and the right anterior periantral fat (arrow head) as well. Based on the imaging signs, the possibility of ROCM was suggested and a prompt CE MRI of the neck was performed. Unenhanced axial T1W (**b**) and coronal T2-FS (**c**) images of the neck confirm the presence of soft tissue in the right PPF (thick arrows in **b**, **c**), right retro-antral fat (thin arrow in **b**) and right anterior periantral fat (arrow head in **b**). CE FS axial T1W image (**d**) reveals heterogeneous enhancement of the soft tissue in the right PPF (thick arrow) as well as in the right retro-antral fat (thin arrow) and right anterior periantral fat (arrow head)

**Fig. 10:**
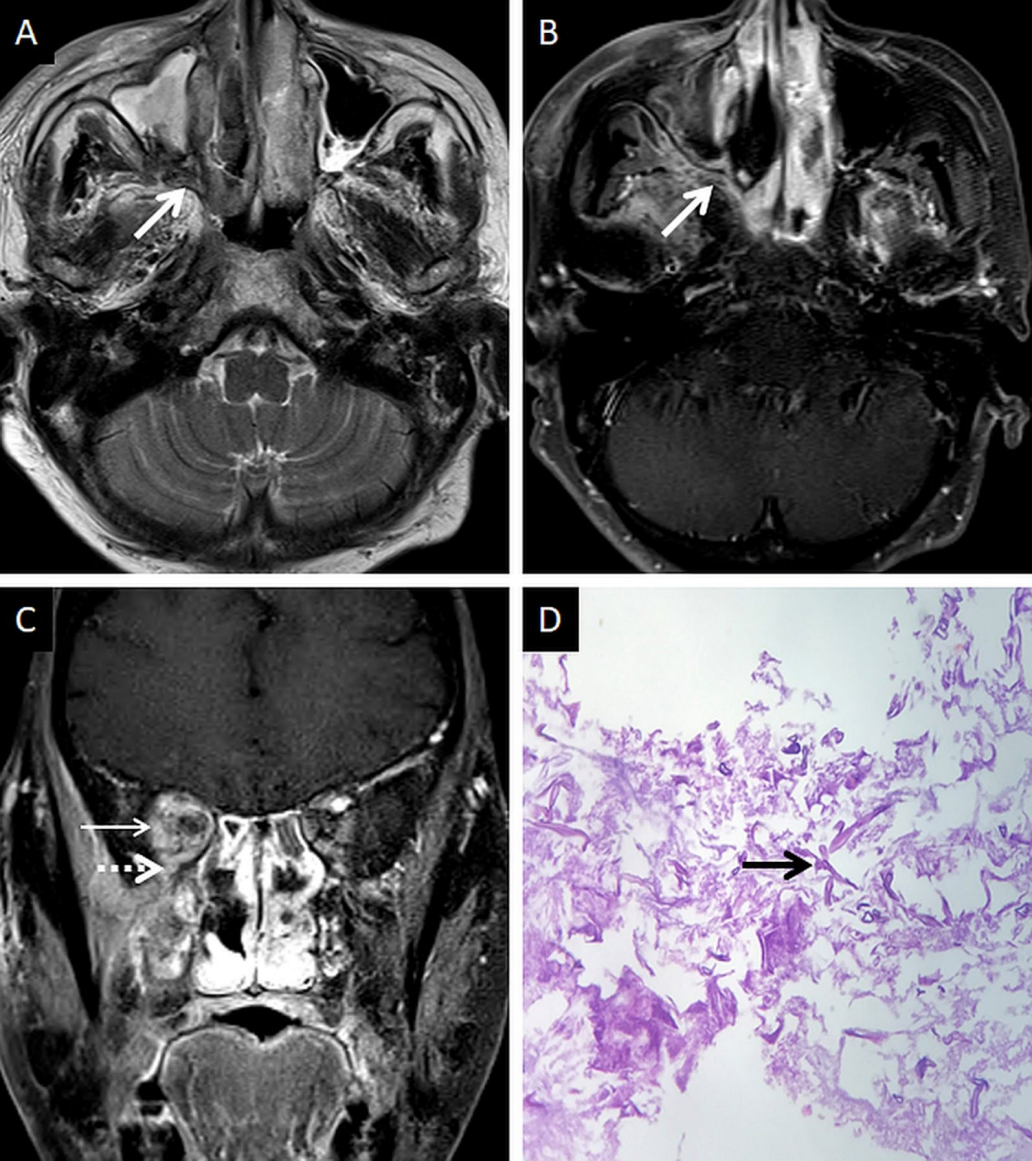
PPF involvement with orbital spread of ROCM. Axial T2W image of the brain (**a**) reveals hypointense soft tissue within the right PPF (thick arrow). CE FS axial T1W image (**b**) demonstrates heterogeneous enhancement of the soft tissue (thick arrow). CE FS T1W image (**c**) reveals ipsilateral intraorbital extension (thin arrow) of the soft tissue, via the inferior orbital fissure (dotted arrow). Post-debridement histopathology analysis (**d**) confirms branching fungal hyphae (thick arrow) compatible with the diagnosis of MCR

Normally, fat is seen around the branches of the internal maxillary artery in the PPF [36]. Obliteration of this fat must raise suspicion for extension of the infection [36] (Fig. 9). In our literature search, involvement of the deep neck spaces, including the PPF, have only occasionally been alluded to. In a case series of 10 patients with ROCM, involvement of the PPF was seen in all patients. Accounting for this, the authors suggested routine exploration of the PPF during surgical debridement in patients with ROCM [39]. Owing to the aggressive spread of MCR and the extensive communication of the PPF with other vital structures (oral cavity, orbit, middle cranial fossa), we recommend inclusion of dedicated neck imaging studies in all patients with suspected ROCM coupled with a thorough evaluation of the PPF in each case (Fig. 10).

**Fig. 11:**
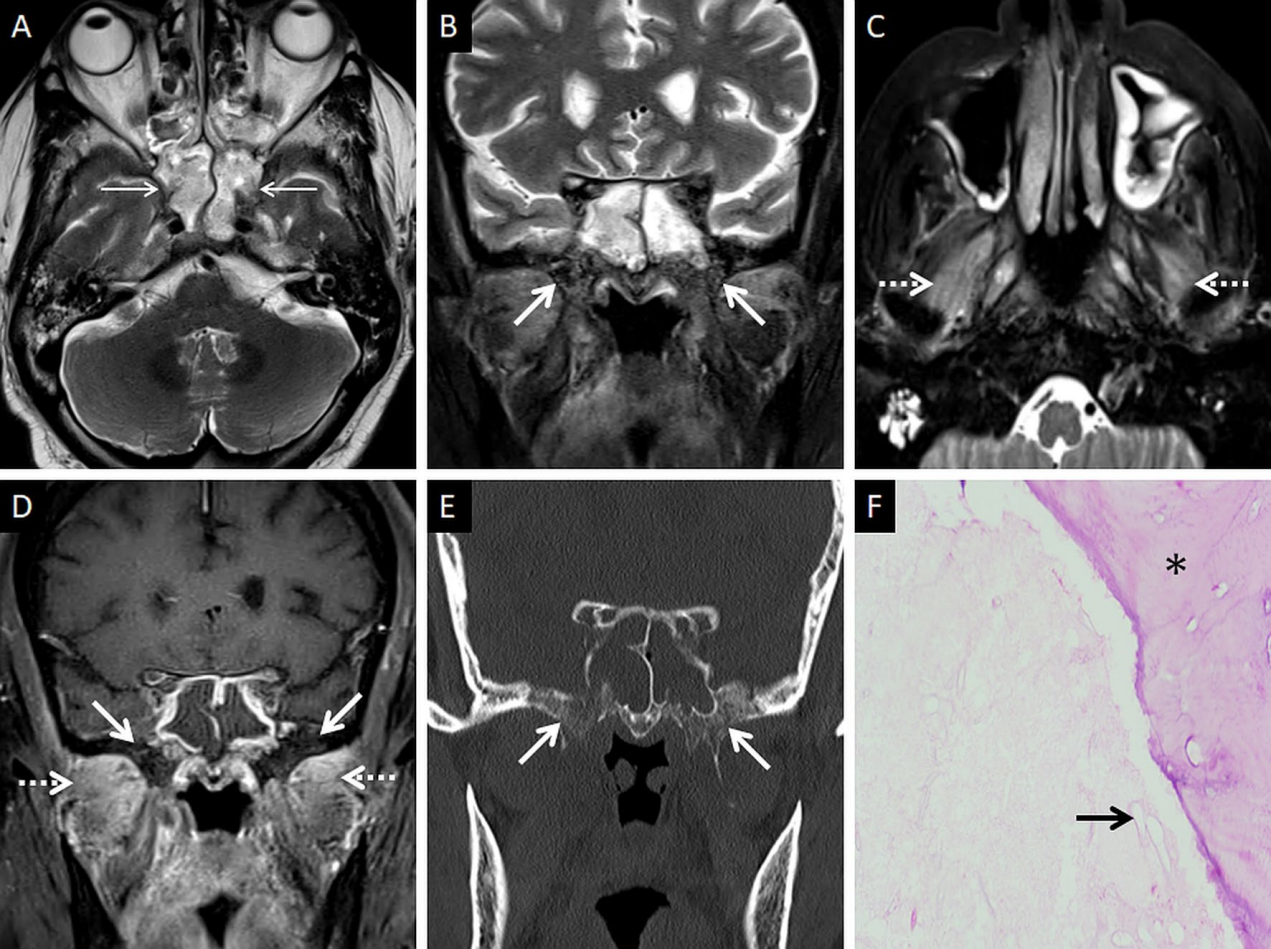
SBO in ROCM. Axial T2W image of the brain (**a**) reveals bilateral sphenoid sinusitis (thin arrows). Coronal T2-FS image of the neck (**b**) demonstrates abnormal hyperintense marrow signals along the base of the sphenoid sinus and the greater wings of the sphenoid bone (thick arrows). Axial T2-FS image (**c**) shows oedematous swelling of the muscles of mastication (dotted arrows). CE FS coronal T1W image (**d**) demonstrates subtle abnormal marrow enhancement within the sphenoid bones (thick arrows) along with intense enhancement of the muscles of mastication (dotted arrows). Coronal CT image (**e**), using bone algorithm, confirms rarefaction of the sphenoid bones (thick arrow). Post-operative histopathology analysis (**f**), using HE staining, confirms Mucorales hyphae (thick arrow) with bone devitalisation and necrosis of the sphenoid bone (*)

In furtherance of the review of the deep neck spaces, we also suggest evaluation of the skull base. Skull base osteomyelitis (SBO) is rare in ROCM, even in the presence of deep neck involvement [22]. SBO in ROCM has been recorded in the chronic phase of the infection [2, 22]. However, we encountered one patient presenting with acute cranial nerve palsies with radiological evidence as well as pathologically proven SBO (Fig. 11). Marrow signal alterations, seen as hypointense on T1W and hyperintense on STIR images, are features typical of SBO [22, 41]. MCR-related SBO may reveal abnormal heterogeneous marrow enhancement with non-enhancing areas representing devitalised bone on contrast-enhanced (CE) MRI [41].

**Fig. 12:**
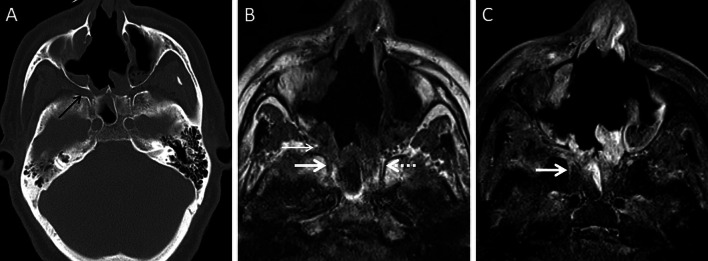
Perineural spread of ROCM detected on post-operative follow-up imaging. Axial CT image (**a**), obtained using bone algorithm, reveals slight widening of the right PPF (thin arrow). Unenhanced axial T1W image (**b**) of the same patient confirms soft tissue (thin arrow) within the right PPF. Note the loss of normal fat signal within the right vidian nerve canal (thick arrow), compared to the contralateral side (dotted arrow). CE FS axial T1W image (**c**) reveals abnormal enhancement of the soft tissue within the right vidian nerve canal (thick arrow). The diagnosis of perineural invasion of MCR was suggested. There was no obvious enhancement along the greater superior petrosal nerve detected at the time of scanning

A thorough evaluation of the deep neck spaces and the skull base also entails evaluation of the peripheral nerves and skull base foramina [42]. Perineural involvement in ROCM constitutes an important pathway of intracranial spread. In a retrospective histopathological review of 11 patients with ROCM, 8 out of 9 specimens with neural tissue showed evidence of perineural spread [43]. In another pathological analysis of ROCM, 19 biopsied peripheral nerves yielded evidence of perineural spread in 15 samples (72.1%) [44]. The cause perineural spread of MCR is unknown, but thought to be related to a favourable intraneural microenvironment and neurotropic factors (secreted within the nerves) [44]. Only a limited number of cases have reported radiological evidence of perineural spread, the trigeminal nerve being most commonly involved [42, 45, 46]. With the sparse reported radiological data, it can be assumed that perineural spread shows imaging patterns similar to head and neck malignancies, including obliteration of perineural fat pads, nerve enhancement and widening of the skull base foramen [46, 47] (Fig. 12).

### Orbital involvement

Orbital involvement results from spread of MCR via the nasolacrimal duct and lamina papyracea [23]. Spread may also occur from the PPF via the inferior orbital fissure [39]. Independent studies on ROCM, yielded an incidence of orbital invasion ranging between 76 and 80% [2, 23].

**Fig. 13:**
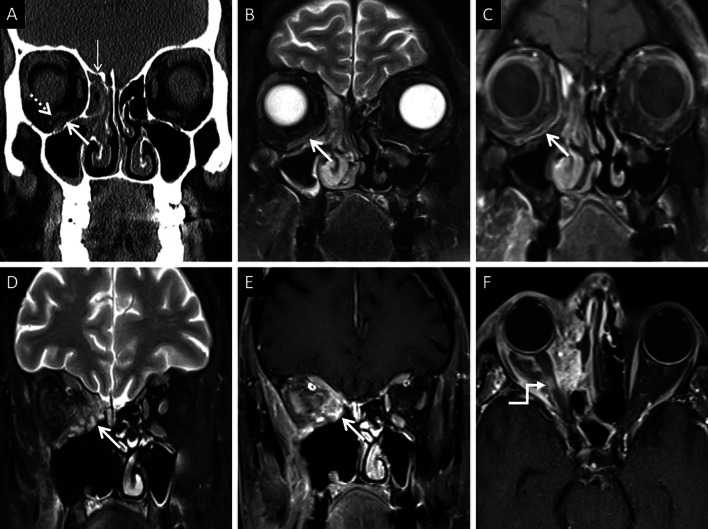
Orbital spread in a patient with histopathologically and microbiologically confirmed ROCM. Coronal CT image of the paranasal sinuses (**a**), obtained using the bone algorithm, reveals right ethmoid sinusitis (thin arrow). Note the subtle extra-conal fat stranding (thick arrow) and mild displacement of the right inferior rectus (dotted arrow). Based on the clinical suspicion of ROCM and CT findings a CE MRI was suggested. Coronal T2-FS image (**b**) confirms the soft tissue within the right extra-conal space (thick arrow), detected previously on the CT. CE FS coronal T1W image (**c**) demonstrates heterogeneous enhancement of the soft tissue (thick arrow). The patient underwent surgical debridement along with right turbinectomy and removal of the right lamina papyracae, followed by a post-operative CE MRI (**d**–**f**) follow-up. Coronal T2-FS (**d**) reveals worsening of the disease with a confluent soft tissue (thick arrow) involving the orbital fat and the extra-ocular muscles. CE FS coronal T1W image (**e**) reveals intense enhancement of the right orbital soft tissue (thick arrow). CE FS axial T1W image (**f**) reveals focal fungal invasion of the right optic nerve (stepped arrow)

Orbital soft tissue, similar to PNS contents, is a typical imaging feature of intraorbital MCR spread [2]. Orbital fat stranding, including the retro-bulbar region, as well as thickening and/or displacement of the medial rectus (indicating spread from ethmoid sinuses) are also frequently encountered [22, 48] (Fig. 13). Proptosis may occur due to retro-bulbar inflammation and/or orbital apex involvement [22, 39]. Orbital apex involvement occurs due to spread from the sphenoid sinuses and forms an important conduit for intracranial spread [49]. MCR is the most common aetiology of orbital apex syndrome among all fungal infections [49].

**Fig. 14:**
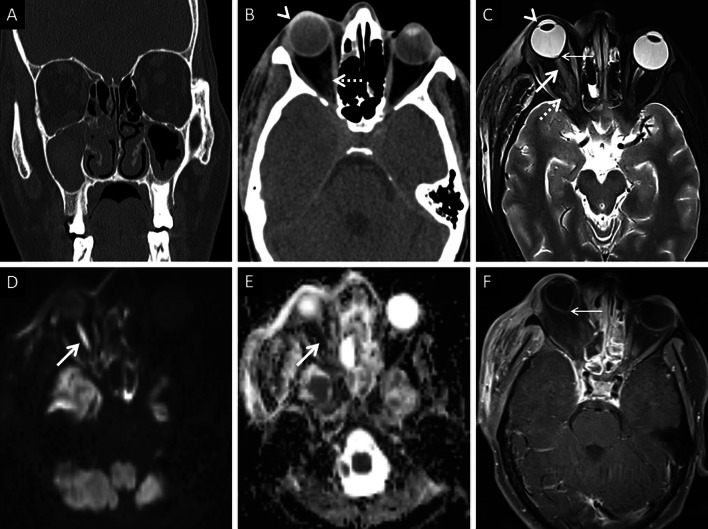
ROCM-related ONI and endophthalmitis. Coronal CT image of the paranasal sinuses (**a**), obtained using the bone algorithm, reveals opacification of the maxillary sinuses, worse on the right. Axial CT image (**b**), using the soft tissue algorithm, demonstrates subtle right orbital fat stranding (dotted arrow) and mild right proptosis (arrow head). Axial T2-FS image (**c**) confirms the right orbital stranding (dotted arrow) and right proptosis (arrow head). Note the subtle tenting of the posterior aspect of the right globe (thin arrow) and thickening of the right optic nerve (thick arrow). Axial diffusion weighted (**d**) and apparent diffusion coefficient (**e**) images reveal restricted diffusion within the right optic nerve in keeping with an optic nerve infarction. Overall, findings of orbital compartment syndrome with endophthalmitis and optic nerve infarction was suggested. The patient underwent urgent surgery, with removal of the right lamina papyracae, for relief of the compartment syndrome. CE FS axial T1W image (**f**) reveals interim worsening of the right endophthalmitis (thin arrow)

Occlusion of the orbital vessels (superior ophthalmic vein, ophthalmic artery), due to MCR-related vasculitis and thrombosis, may also be noted [22]. The occlusion of the ophthalmic artery and/or central retinal artery may result in optic nerve infarction (ONI). In our literature review, we found 7 previously published reports of ONI in patients with ROCM [50–56]. Of these, there were 2 reports of bilateral ONI and 1 report of ONI being the initial presentation of orbital MCR [51, 52, 55]. Infarcted optic nerves demonstrates restricted diffusion and normal FLAIR signals on MRI [51] (Fig. 14).

### Intracranial spread

#### Neuroparenchymal involvement

The anterior cranial fossa is the typical site of MCR involvement. Spread occurs through erosions of the cribriform plate or via perineural spread along the olfactory nerves [57]. The olfactory nerves, with their projections into the paleocortex (basifrontal lobe) and the neocortex (mesial temporal lobe), serve as important conduits for preferential involvement of these region [58]. Involved olfactory nerves appear thickened and hypointense on T2W images [58].

**Fig. 15:**
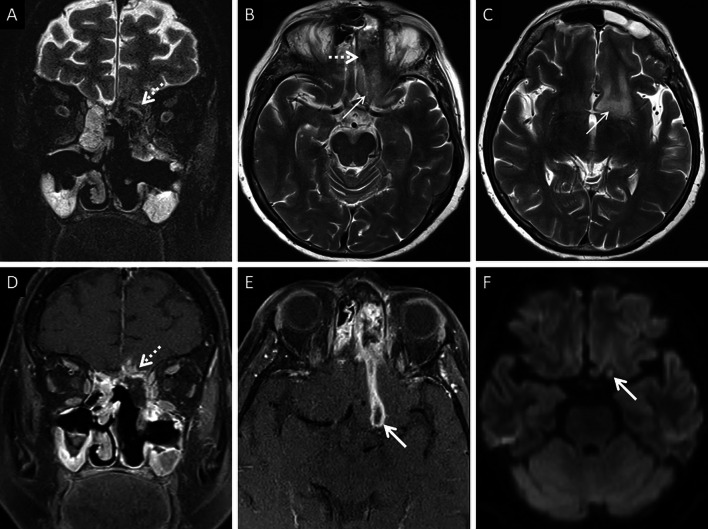
Cerebritis and parenchymal abscess formation in ROCM. Pre-operative imaging workup was performed at another institution. Post-operative MRI, performed at our institution, is presented. Coronal T2-FS image (**a**) demonstrates ill defined, hypointense soft tissue in the left ethmoid air cells, invading into the left anterior cranial / left olfactory fossa (dotted arrow). Axial T2W images of the brain (**b**, **c**) demonstrates the hypointense soft tissue (dotted arrow in B) along with vasogenic oedema in the left basifrotnal white matter (thin arrows in **b**, **c**). CE FS coronal T1W image (**d**) shows abnormal parenchymal enhancement (dotted arrow) of the left gyrus rectus suggesting cerebritis, due to fungal invasion. CE FS axial T1W image (**e**) shows a peripherally enhancing lesion (thick arrow) along the posterior portion of the left gyrus rectus. Axial diffusion weighted image (**f**) shows restricted diffusion within the peripherally enhancing lesion (thick arrow), suggesting abscess formation

Neuroparenchymal infection begins with cerebritis, seen as cortical/subcortical areas of T2/FLAIR hyperintensity with or without contrast enhancement. This stage may eventually progress to a peripherally enhancing abscess with contents demonstrating restricted diffusion and T2 hypointensity (attributable paramagnetic fungal material) [57] (Fig. 15). MR spectroscopy may help in narrowing down to a diagnosis; elevated lactate and trehalose (3.8 ppm) have been described [59, 60]. The lack valine and isoleucine (0.9 ppm) and leucine (3.6 ppm), seen in the bacterial abscesses, may help guide the radiologist towards MCR [59]. MR spectroscopy however needs further validation with a larger case-based series.

#### Neurovascular complications

Vascular involvement is the most dreaded complication of MCR due to the associated poor prognosis. In a meta-analysis performed by Mazzai et al., 42 cases of MCR-related vascular complications were identified between 1975 and 2019. Outcome of their research revealed 35 cases of arterial and/or cavernous sinus thrombosis with cerebral ischaemia; 6 cases of subarachnoid haemorrhage; 5 cases of arterial aneurysms; and 1 case of septic emboli. Of these, only 9 patients survived [57].

**Fig. 16:**
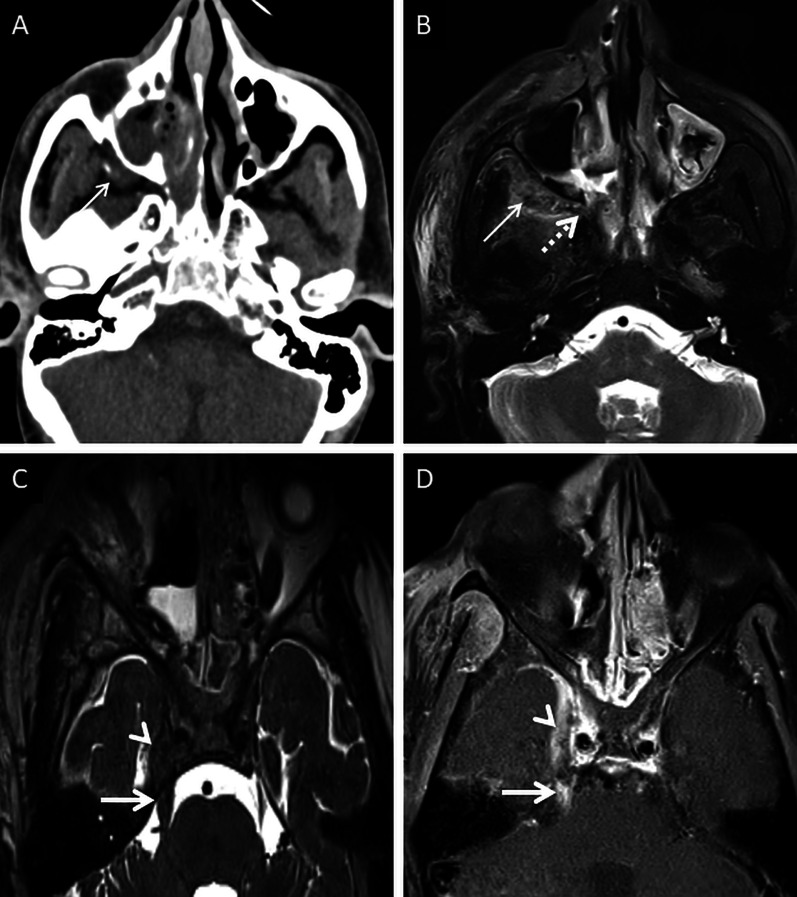
Post-operative follow-up demonstrating intracranial involvement in a case of ROCM. Axial CT image (**a**) of the neck reveals mild soft tissue in the right retro-antral (thin arrow). Axial T2-FS image (**b**) confirms the soft tissue occupying the right retro-antral fat (thin arrow) and PPF (dotted arrow). Axial constructive interference in steady state (CISS) sequence (**c**) obtained for the evaluation of the cranial nerves demonstrates thickening of the right trigeminal nerve (thick arrow). Note the lateral convexity of the right cavernous sinus (arrow head). CE FS T1W image (**d**) reveals dural thickening and non-enhancement of the right cavernous sinus in keeping with thrombosis (arrow head). Enhancement of the cisternal portion of the right trigeminal nerve (thick arrow in **d**) is also seen, suggestive of intracranial perineural spread

Cavernous sinus thrombosis is common, occurring due to spread of MCR from the orbit, across the orbital apex [23]. On MRI, a thrombosed cavernous sinus demonstrates loss of flow voids on T1W and T2W images with a convexity along its lateral margin. CE examinations reveal filling defects within an otherwise enhancing sinus [23, 57] (Fig. 16). Intracranial spread may also result in occlusion of the surface veins and dural venous sinuses (especially the superior sagittal sinus). Such occlusions may result in venous ischaemia with/without haemorrhagic conversion. “Blooming” on gradient echo or susceptibility weighted images SWI must raise concern for a venous occlusion and prompt confirmation with a venogram study [57, 61].

Arterial occlusions may secondary to cavernous sinus invasion/thrombosis (internal carotid artery) and leptomeningeal disease or due to direct spread from the sphenoid sinus (basilar artery) [57]. The high propensity for arterial invasion by MCR is due to its ability to reproduce in the internal elastic layer of the vessel, causing separation of the elastic and middle layers leading to intimal damage and thrombosis [62]. Ma et al. also report purulent arteritis and inflammatory emboli caused by arterial invasion by MCR, thus leading to cerebral infarction or mycotic aneurysm [63].

**Fig. 17:**
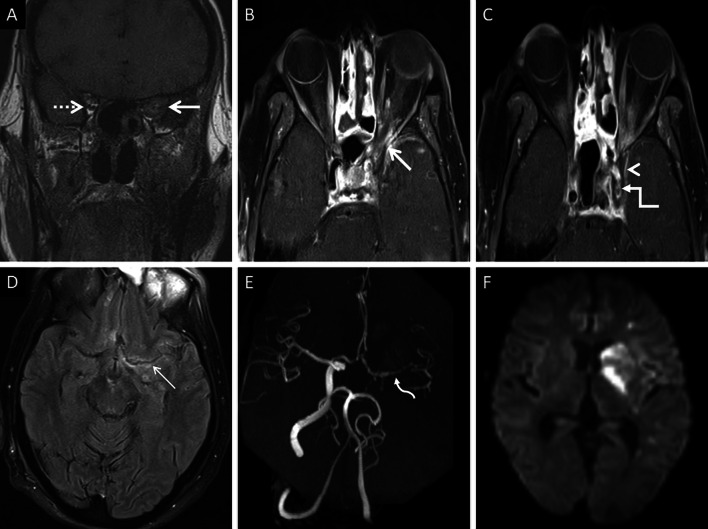
Intracranial spread of ROCM in a patient with bilateral ethmoid sinus disease (not shown). Coronal T1W image (**a**) reveals abnormal soft tissue within the left orbital apex (thick arrow). Note the normal fat signals in the contralateral orbital apex (dotted arrow). CE FS axial T1W images (**b**, **c**) demonstrate the soft tissue in the left orbital apex (thick arrow in **b**), non-enhancement of the left cavernous sinus (arrow head in **c**) suggesting thrombosis, and loss of flow void along the left carotid siphon (stepped arrow in **c**) representing occlusion. Axial CE FLAIR image (**d**) reveals leptomeningeal enhancement along the left middle cerebral artery sulcus (thin arrow). 3-D reconstruction of a Time-of-Flight MR angiogram (**e**) confirms the occlusion of the left internal carotid artery along with attenuated, irregular flow signals within the M1 segment of the middle cerebral artery (curved arrow), reformed via the circle of Willis, and reduced distal cortical arborisation. Diffusion weighted image (**f**) demonstrates scattered acute infarcts in the left middle cerebral artery territory. In summary, the diagnosis of ROCM with orbital apex involvement and intracranial spread (cavernous sinus thrombosis, leptomeningeal disease and MCR-related vasculitis) was suggested

Unenhanced CT in an acute setting or diffusion weighted imaging help in identifying neuroparenchymal infarction. Loss of arterial flow voids on T1W and T2W images must raise the suspicion for a thrombo-occlusion. Intra-arterial hypointensity on SWI suggests the presence of a thrombus. CT or MR angiograms can help detected the site of the occlusion. MRI vessel wall imaging may reveal abnormal enhancement of the arterial wall with a non-enhancing intraluminal thrombus [57] (Fig. 17).

It must be noted that vascular complications of MCR are not limited to thrombo-occlusions [50]. Mycotic aneurysms are associated with an increased mortality, and hence, an active, thorough search must be performed on angiogram studies (57). Rupture of mycotic aneurysm results in subarachnoid haemorrhage, seen as hyperdensity and susceptibility in the CSF spaces on CT and SWI imaging, respectively.

## Conclusion

ROCM is a common cause of IFS in diabetics and immunocompromised patients. Since 2020, ROCM co-infection has been identified in COVID-19 patients, challenging patient management. ROCM presents with a fulminant clinical course, needing prompt and aggressive treatment. Imaging provides rapid diagnosis and plays a crucial role in delineating spread of the disease, thus guiding surgical interventions. The BT sign is a common finding in most patients with ROCM, but it may be seen in other IFS and may also be mimicked by normal cavernous tissues in the turbinates. The angioinvasive property of the fungus facilitates spread beyond the sinus without overt bone destruction. Thus, extension of MCR into the neck spaces and the orbits occurs early in the disease; fat stranding may be the only imaging evidence of extra-sinus spread. Hence, close evaluation of soft tissue algorithms on CT and fat-suppressed sequences on MRI is imperative in detection. Additional detailed imaging of the base of skull must be considered in suspected cases of MCR as the fungus has the potential to spread along peripheral nerves in the base of the skull into the intracranial compartment. Intracranial manifestations of the infection vary; ranging from abscess formation to vasculitis-related ischaemic complications, all of which are harbingers of a poor outcome. Knowledge of these imaging manifestations with an algorithmic approach in evaluation of disease ensures a thorough evaluation of each case which impacts patient care and outcome.

## Data Availability

All data are included in this manuscript.

## Abbreviations

BT: Black turbinate
CAM: Coronavirus disease 2019 (COVID-19)-associated Mucormycosis
CE: Contrast-enhanced
COVID-19: Coronavirus disease 2019
DM: Diabetes mellitus
FS: Fat-suppressed
HE: Haematoxylin and Eosin
IFS: Invasive fungal sinusitis
MCR: Mucormycosis
ONI: Optic nerve infarction
PPF: Pterygopalatine fossa
ROCM: Rhino-orbito-cerebral Mucormycosis
SBO: Skull base osteomyelitis
